# Editorial: Immunonutrient Supplementation

**DOI:** 10.3389/fnut.2019.00182

**Published:** 2019-12-03

**Authors:** Thea Magrone, Alexander Haslberger, Emilio Jirillo, Mauro Serafini

**Affiliations:** ^1^Department of Basic Medical Sciences, Neuroscience and Sensory Organs, School of Medicine, University of Bari, Bari, Italy; ^2^Department of Nutritional Sciences, University of Vienna, Vienna, Austria; ^3^Functional Foods and Metabolic Stress Prevention Laboratory, Faculty of Bioscience and Technology for Food, Agriculture and Environment, University of Teramo, Teramo, Italy

**Keywords:** polyphenols, fatty acid, vitamins, amino acids, iron

Nowadays, food-related diseases are exponentially increasing worldwide. In fact, also in developing countries population has started consuming western diet. On these bases, overweight/obesity and diabetes represent a pandemy, thus, leading to associated diseases such as cardiac events, neurodegeneration, and cancer (1–3). Correct diets, e.g., Mediterranean diet (MeD), as well as nutrient supplementation represent a suitable approach to prevent and/or mitigate food-related diseases. In particular, among bioactive principles contained in foods, mostly polyphenols, have been investigated for their anti-oxidant, anti-inflammatory activities, even including DNA damage protection (4–9).

The present special issue entitled “Immunonutrient supplementation” encompasses a series of reviews and original articles which point out the modulatory effects of diet and of different nutrients (polyphenols, amino acids, unsaturated fatty acids, vitamin D, and iron, respectively) on the immune response.

Ruiz-Leòn et al. have reviewed the principles of immune nutrition in relation to atherosclerosis development. MeD, for its content in bioactive compounds, has been associated to prevention or attenuation of atheroma inflammation. Here, Authors have analyzed the molecular mechanisms related to the *in vivo* protective actions of MeD.

Wu et al. have reviewed the efficacy of interventions with unsaturated fatty acids, micronutrients, functional foods and tea derivatives on the immune function. Despite controversial results, there is strong evidence that all above mentioned principles play a protective role in autoimmune and inflammatory disorders also reducing infections.

Campbell et al. have reported the effects of two polyphenols, carnosol and curcumin, on the metabolism of dendritic cells (DCs). Metabolic regulation of DCs exerted by these polyphenols seems to be related to AMP-Activated Protein Kinase (AMPK) activation, which leads to the inhibition of mTOR pathway in lipopolysaccharide-primed DCs. In addition, activation of AMPK induces Heme Oxygenase-1 (HO-1), which, in turn, controls maturation, and function of human DCs.

Zhang et al. have studied the effects of Inonotus sanghuang polyphenols on the interaction between macrophages and adipocytes. Results show that these polyphenols are able to decrease chronic inflammation in adipose tissue, suppressing the cross talk between macrophages, and adipocytes.

Tuyaerts et al. have reported the effects of dietary curcumin (2 g/day for 2 weeks) in seven endometrial carcinoma (EC) patients. During this clinical trial, several inflammatory biomarkers were measured, even including COX-2 and frequency of T cells, DCs, natural killer cells and myeloid-derived suppressor cells. In addition, quality of life (QoL) questionnaires were completed by patients at the start and the end of treatment. Authors conclude that this treatment does not lead to significant modifications of all parameter analyzed as well as of QoL.

Wang et al. have evaluated the effects of naringenin, a polyphenol, on CD4+ T cells in a model of experimental autoimmune encephalomyelitis (EAE). Among major effects exerted by naringenin, inhibition of differentiation of CD4+ cells to Th1 and Th17 cells, decrease of Th17 cells and promotion of T regulatory (Treg) cell polarization have been reported. Then, all these activities would explain the beneficial effects of naringenin in preventing/mitigating EAE.

Azam et al. have reviewed the effects of polyphenols in the treatment of neurodegenerative diseases. Special emphasis has been placed on the ability of various polyphenols to dampen over-expression of inflammatory mediators interrupting the Toll-like receptor (TLR)-4/NF-κB/STAT pathway in microglia and macrophages. In the same direction, other polyphenols can decrease neuronal apoptosis, regulating the TLR-4/MyD88/NF-κB signaling. Therefore, modulation of TLR functions by polyphenols may represent a novel therapeutical tool in the treatment of neurodegeneration.

Lee et al. have reported on the effects of L-arginine and L-citrulline supplementation on rat Treg cells. Male infantile rats received L-arginine or L-citrulline (200 mg/kg/day *i.p*.) over postnatal day 8 to day 14. Both amino acids increased interleukin (IL)-10 release, while enhancing SIRT-1 expression. Only in the case of L-citrulline increase in the transforming growth factor (TGF)-β1 and FoxP3 expression was noted. In conclusion, these amino acids have the ability to induce a tolerogenic pathway, thus, favoring anti-inflammatory activities under pathological conditions.

Zhang et al. have evaluated the effects of dietary L-tryptophan treatment on the Chinese mitten crab, *Eriochier* (*E*.) *sinensis* under cheliped autotomy stress. In treated individuals, mortality decreased with an increase in total hemocyte count, hemocyanin and glutathione (GSH) content and GSH peroxidase. Furthermore, increase in phagocytic rate and anti-oxidant activity was observed. These data were in agreement with the higher expression of anti-bacterial related protein genes. Taken together, these results indicate the ability of polyphenols to increase survival of *E*. *sinensis* under cheliped autotomy stress.

Machado et al. have demonstrated the ability of dietary methionine to improve immune and inflammatory responses and disease resistance in European sea bass (*Dicentrarchus labrax*). Then, following bacterial challenge a higher survival was observed in comparison to untreated fish, thus, suggesting a reinforcement of immune response after 4 weeks of methionine treatment, thus, supporting the anti-bacterial activity.

Xia et al. have summarized the mechanisms of betaine in IL-1β production release. This compound, which is a critical nutrient for mammal health, inhibits IL-1β release blocking exocytosis of IL-1β containing secretory lysosomes, thus, reducing shedding of IL-1β containing plasma membrane microvesicles. Then, these mechanisms reduce the passive efflux of IL-1β through plasma membrane in the course of pyroptotic cell death, representing a therapeutical tool in the course of IL-1β associated-inflammatory disease.

Saika et al. have reviewed the effect of lipid metabolites in the host and the participation of intestinal bacteria in this process. In particular, the role of metabolites from omega-3 fatty acids, such as resolvins, protectins, and marensins has been described. Special emphasis has been placed on the metabolites of 17, 18-epoxyeicosatetraenoic acid, which exert anti-allergic and anti-inflammatory activities.

Goncalves-Mendes et al. have conducted a trial investigating the effects of vitamin D supplementation on deficient elderly persons in relation to influenza vaccination. Vitamin D supplementation has been shown to trigger elevated plasma levels of TGF-β in response to influenza vaccination, thus, polarizing the immune response toward a tolerogenic pathway. No enhancement of antibody production to influenza vaccine was observed.

Dufrusine et al. have analyzed the influence of iron on the 5-lipoxygenase (LOX) trafficking and human macrophage activation. Results indicate that iron regulates the biological activity of 5-LOX in macrophages, enhancing its ability to bind to nuclear membranes. In relevance to this effect, iron overloading induced an increased expression of IL-6 in macrophages which was abolished by pre-treating cells with the iron-chelating agent deferoxamine.

As overall, the manuscripts have discussed different aspects related to immunonutrient supplementation, ranging from *in vitro, ex vivo* and human studies, highlighting the complexity, and the multi-faced aspects of the interactions between nutrient supplementation and immune function. Despite more research is needed, we think that this special issue should allow readers to increase their knowledge on the beneficial and sometimes adverse effects related to the role of nutrient supplementation on immune function.

## Author Contributions

All authors listed have made a substantial, direct and intellectual contribution to the work, and approved it for publication.

### Conflict of Interest

The authors declare that the research was conducted in the absence of any commercial or financial relationships that could be construed as a potential conflict of interest.
